# Characterization of *Mycobacterium tuberculosis *Beijing isolates from the Mediterranean area

**DOI:** 10.1186/1471-2180-10-151

**Published:** 2010-05-25

**Authors:** M Alonso, N Alonso Rodriguez, C Garzelli, M Martínez Lirola, M Herranz, S Samper, MJ Ruiz Serrano, E Bouza, D García de Viedma

**Affiliations:** 1 Servicio de Microbiología Clínica y Enfermedades Infecciosas, Hospital Gregorio Marañón, Madrid, Spain; 2 Unidad Central de Análisis Molecular (UCAM), Hospital Gregorio Marañón, Madrid, Spain; 3 CIBER Enfermedades Respiratorias (CIBERES), Spain; 4 Dipartimento de Patologia Sperimentale, Biotecnologie Mediche, Infettivologia ed Epidemiologia, Università de Pisa, Pisa, Italy; 5 Complejo Hospitalario Torrecárdenas, Almería, Spain; 6 Instituto Aragonés de Ciencias de la Salud, Zaragoza

## Abstract

**Background:**

The Beijing lineage of *Mycobacterium tuberculosis *is causing concern due to its global distribution and its involvement in severe outbreaks. Studies focused on this lineage are mainly restricted to geographical settings where its prevalence is high, whereas those in other areas are scarce. In this study, we analyze Beijing isolates in the Mediterranean area, where this lineage is not prevalent and is mainly associated with immigrant cases.

**Results:**

Only 1% (N = 26) of the isolates from two population-based studies in Spain corresponded to Beijing strains, most of which were pan-susceptible and from Peruvian and Ecuadorian patients. Restriction fragment length polymorphism typing with the insertion sequence IS*6110 *identified three small clusters (2-3 cases). Mycobacterial interspersed repetitive unit-variable number tandem repeat typing (MIRU-15) offered low discriminatory power, requiring the introduction of five additional loci. A selection of the Beijing isolates identified in the Spanish sample, together with a sample of Beijing strains from Italy, to broaden the analysis context in the Mediterranean area, were assayed in an infection model with THP-1 cells. A wide range of intracellular growth rates was observed with only two isolates showing an increased intracellular replication, in both cases associated with contained production of TNF-α. No correlation was observed between virulence and the Beijing phylogenetic group, clustered/orphan status, or resistance. The Beijing strain responsible for extensive spread on Gran Canaria Island was also identified in Madrid, but did not lead to secondary cases and did not show high infectivity in the infection model.

**Conclusions:**

The Beijing lineage in our area is a non-homogeneous family, with only certain highly virulent representatives. The specific characterization of Beijing isolates in different settings could help us to accurately identify the virulent representatives before making general assumptions about this lineage.

## Background

Tuberculosis (TB) is one of the main infectious causes of death worldwide, with more than 9 million new cases of active disease every year and nearly 2 million deaths [1]. *Mycobacterium tuberculosis *(MTB) is the causative agent of most TB cases, and its ability to spread and the outcome of infection depend on epidemiological, host, and bacterial factors [2].

The MTB genome is highly conserved, but several large sequence polymorphisms defining different genetically related lineages have been identified. Among them, the Beijing family can be identified rapidly and reliably by several genetic features. These include a characteristic spoligotype with exclusive deletion of spacers 1-34 (the so-called RD207 deletion) [3], an intact open reading frame in the *pks15/1 *gene [4], and deletion of the genomic region RD105, which define the Beijing family as a separate lineage within MTB [5].

The Beijing lineage is causing major concern worldwide [6,7] because its worldwide spread and involvement in several TB outbreaks, some of them involving drug-resistant strains [8]. The Beijing lineage is generally considered to be associated with drug-resistance, although this association has not been found in all geographic settings [7,8]. The proportion of Beijing strains differs, being low in Western Europe, although a slight increase in the number of Beijing strains has been detected over time [6]. The presence of this lineage in the population has been associated with the recent increase in the number of TB cases among immigrants observed in several areas, including the Mediterranean [8,9].

The wide distribution of Beijing strains suggests that members of this phylogenetic lineage are better adapted to infect and cause disease in humans than other MTB families, and there are reports indicating that Beijing strains show higher replication rates and more virulent phenotypes than other MTB lineages in both *in vitro *and *in vivo *models [10,11]. The infective success of this lineage seems to be associated with its effect on the immune response, in that it can control the release of the macrophage-derived cytokines that play a central role in directing the immune response towards a non-protective Th2 phenotype [12,13].

The incidence of the Beijing lineage in Spain is low, although in recent years it has been increasing due to immigration [9]. The profile of nationalities of the immigrants infected by Beijing isolates differs from that observed in other countries, and South American cases are the most common. The impact of the importation of Beijing isolates to Spain was described in the 1990s on Gran Canaria Island, where an extensive outbreak involving this lineage was detected after a Beijing isolate was identified in an immigrant [14].

Studies analyzing the Beijing lineage are scarce in the Mediterranean area [15,16]. We explored whether specific genotypic and phenotypic features could be found for the Beijing strains isolated in a context where this clade is not endemic, but imported by immigrants whose origin (mainly Peru and Ecuador) is different from that found in other settings.

## Results

### Identification and characterization of Beijing isolates

Of the 2391 isolates analyzed in the Spanish sample, 26 (1.09%) were identified as members of the Beijing lineage according to the criteria reported in the Methods section. In particular, nineteen showed deletion of the spacers 1-34 and the characteristic hybridization pattern of spacers 35-43, and the remaining seven corresponded to variant "Beijing-like" spoligotypes.

In order to verify the spoligotyping-based identification of Beijing strains and to refine the genetic characterization, the *pks15/1 *gene and the RD105, RD181, RD150, and RD142 were analyzed. The *pks15/1 *gene, which is generally considered a marker for *M. tuberculosis *strains of Asian origin [4,17], was sequenced in all 26 isolates in order to rule out deletions, and in all cases this gene was intact (Table 1). The genomic deletion RD105, which phylogenetically defines the Beijing family [5], was found in all 26 (Table 1). On the basis of the polymorphisms associated with genomic deletions RD181, RD150, and RD142, previously defined for the Beijing lineage by Reed *et al*[18], all of the isolates belonged to phylogenetic group 3 except one, which belonged to group 4.

**Table 1:** Epidemiological and genetic features of Beijing strains

Total	Nationality	Drug susceptibility^a^	pks15/1 gene^b^	RD105^c^
8	Ecuador	S	+	-
3	Peru	1 INH-R/2 S	+	-
2	Morocco	1 INH-R/1 S	+	-
4	China	S	+	-
1	Armenia	S	+	-
1	Moldavia	S	+	-
1	Ukraine	MDR	+	-
1	South Africa	S	+	-
1	Russia	S	+	-
4	Spain	S	+	-

^a ^MDR, multidrug-resistant; INH-R, isoniazid-resistant; S, pan-susceptible.

^b ^+ indicates *pks15/1 *gene intact.

^c ^-indicates absence of the RD105 genomic region.

By origin, 22 of the 26 isolates were from foreign-born cases (84.6%) of nine different nationalities, the most frequent being Peruvians and Ecuadorians (42%). The remaining four Beijing isolates corresponded to autochthonous cases (Table 1).

The drug susceptibility tests showed that 23 of the 26 isolates were pan-susceptible, two were isoniazid-resistant, and one was multidrug-resistant (Table 1).

### Genotyping analysis

**Figure 1:**
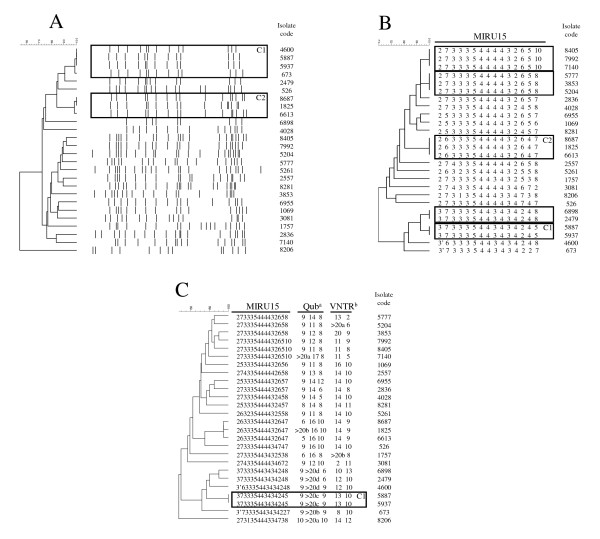
**Comparative analysis of IS*6110*-RFLP (A), MIRU-15 (B), and MIRU-15+5 (C) in the 26 clinical Beijing isolates**. ^a^Order of QUB loci: QUB 11a, QUB 3232, and QUB 18. ^b^Order of VNTR loci: VNTR3820 and VNTR4120. The clustered cases are indicated within boxes. C1 and C2 refer to the cases included in the two clusters defined by RFLP. In some cases, the large size of some products obtained in QUB and the VNTR loci did not allow precise assignation of alleles. In these cases we could only estimate that the number of repetitions was higher than 20 (> 20). When we observed products differing in size in groups of isolates with more than 20 repetitions, we sub-labeled them > 20a, > 20b, > 20c and > 20d.

The IS*6110*-RFLP analysis revealed 21 different genotypes (9-22 IS*6110 *copies). Seven isolates (26.9%) were grouped in two clusters of three and four cases each. Nineteen isolates (73.1%) were unclustered and considered orphan cases (Figure 1A). The isolates involved in cluster 2 (C2) shared an identical IS*6110*-RFLP pattern with those involved in the Gran Canaria outbreak [14].

The MIRU-15 analysis identified 18 different genotypes among the Beijing isolates. Thirteen isolates (50%) were grouped in five clusters of two or three cases. The remaining isolates corresponded to orphan cases (Figure 1B). If we compare RFLP and MIRU-15 data, it is noteworthy that two representatives of cluster 1 (C1), defined by RFLP, were split by MIRU-15, and three of the clusters defined by MIRU-15 grouped isolates that had been considered orphan by RFLP. Only the C2 cluster defined by RFLP remained intact after MIRU analysis. Regarding the isolates clustered in C2, which shared the RFLP pattern with the isolate involved in the Gran Canaria outbreak, we also pursued to compare the MIRU-15 data. With this aim, a selection of Gran Canaria outbreak isolates, sharing also the susceptibility pattern with those form Madrid, were analyzed and an identical MIRU-15 type was shared by the representatives from Madrid and Gran Canaria.

After observing the low discrimination of MIRU-15, five new VNTR loci (QUB11a, QUB3232, QUB18, VNTR3820, and VNTR4120) were added; they were all selected due to their high discriminatory values in different studies focused on Beijing isolates [19,20]. The discriminatory power of this MIRU-15+5 set was markedly higher than that of MIRU-15, and was even higher than that of RFLP. Twenty-five different genotypes were identified from the 26 isolates analyzed. Four of the five MIRU-15 clusters were sub-divided by MIRU-15+5 (Figure 1C), and only the C1 cluster defined by MIRU-15 remained intact.

### Infectivity characterization

#### i) Intracellular growth in THP-1 cells

Eight of the 26 Beijing isolates characterized in the Spanish sample (1-8) were selected to assure a suitable variability according to different features: nationality of the cases (6 nationalities), drug susceptibility (2 resistant and 6 susceptible), number of IS*6110 *copies (9-22) and phylogenetic group (groups 3 and 4) (Table 2). The strain responsible for the outbreak on Gran Canaria Island was also included (isolate no. 1). To widen the geographic setting to the Mediterranean area and to increase both the number of Beijing representatives analyzed and the number of isolates involved in clusters, we included to the Spanish Beijing representatives, eight additional Beijing isolates (9-16) from Tuscany, Italy (Table 2). As controls, we included the virulent reference strain H37Rv and a non-Beijing representative orphan strain.

**Table 2:** Beijing strains assayed in THP-1 cells

Isolate code	Year of isolation	Strain No.	Nationality	Drug susceptibility^a^	IS6110 copy no.^b^	Clustered/Orphan (+/-)^c^	RD Group^d^
8687	2002	1	Spain	S	16	+	3
5204	2005	2	China	S	22	-	3
7992	2005	3	Ecuador	S	20	-	3
8281	2004	4	Armenia	S	21	-	3
6955	2003	5	Moldavia	S	16	-	3
6898	2005	6	Ecuador	S	9	-	3
5261	2006	7	Peru	INH-R	22	-	4
673	2006	8	Ecuador	S	13	+	3
1819	2005	9*	Brazil	S	NA	NA	3
1884	2005	10*	Peru	S	NA	NA	3
1284	2004	11*	Italy	INH-R SM-R	17	+	3
1538	2004	12*	Peru	S	20	+	3
1409	2004	13*	China	S	18	+	3
1254	2003	14*	China	S	21	+	3
838	2002	15*	China	S	11	-	2
1149	2003	16*	Chile	S	9	+	2

^a ^INH-R, isoniazid-resistant; SM-R, streptomycin-resistant; S, pan-susceptible.

^b ^Number of bands identified by RFLP. NA, not available.

^c ^+ and - indicate clustered/orphan status of the strain. NA, not available.

^d ^Phylogenetic classification according to the presence or absence of the RD181, RD150, and RD142 genomic regions, according to Reed *et al *[18].

* Isolates from Tuscany.

**Figure 2:**
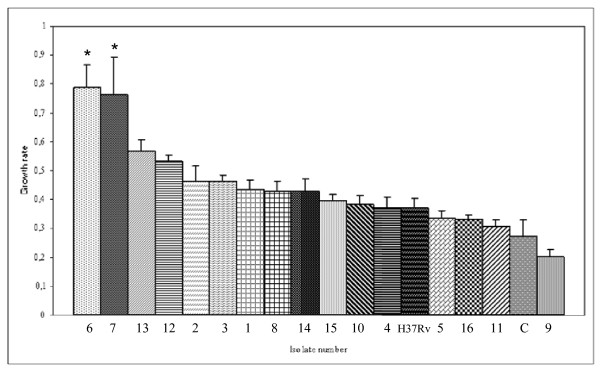
**Intracellular growth rate in differentiated THP-1 cells**. Cells were infected with 16 Beijing isolates, the control strain H37Rv, and a non-Beijing strain (C). Intracellular growth was expressed as the growth rate (Y Axis), ie, the slope of the function of log_10 _CFU values during the infection period. CFUs were determined 3 hours after infection and at days 1, 4, and 7. Results are expressed as the mean ± standard error of the three independent experiments per strain. Asterisks indicate isolates with significantly higher intracellular growth rates (P < 0.05).

A wide range of intracellular growth rates was detected among the Beijing isolates assayed (Figure 2). Two isolates showed the highest intracellular growth rates, which differed significantly (P < 0.05) from the others. There were no significant differences in growth rate among the remaining isolates, including control strain H37Rv and the non-Beijing orphan strain. No correlation was found between the epidemiological status of the isolates (clustered/unclustered) and the intracellular growth rates. The isolate responsible for the outbreak on Gran Canaria Island was included, although it did not show increased intracellular replication.

#### ii) Cytokine production

**Figure 3:**
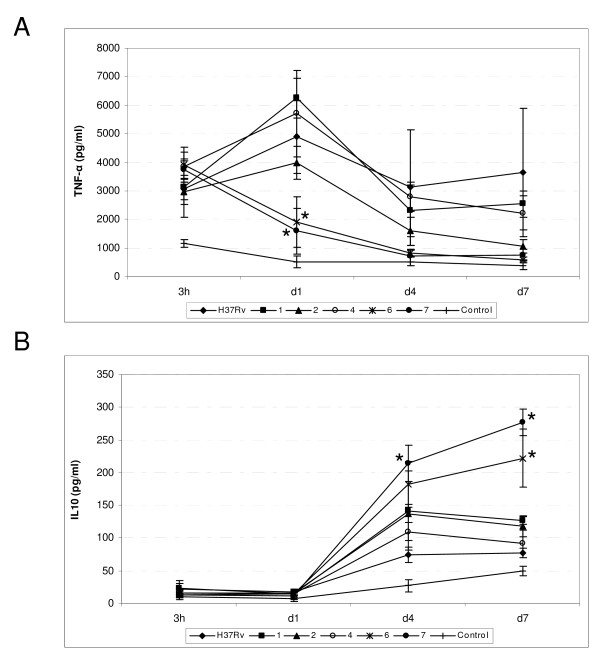
**Cytokine production by differentiated THP-1 cells infected with strains representatives of different intracellular growth rates**. Levels of TNF-α (panel A) and IL-10 (panel B) were determined in culture supernatants 3 hours after infection and at days 1, 4, and 7. Data are expressed as the mean ± standard error of three independent experiments per strain. Asterisks indicate significantly different (P < 0.05) cytokine production. Control: Non-infected control cells.

We studied the immunoregulatory profile of cytokines secreted in THP-1 cells infected with six isolates selected as representatives of the different intracellular growth rates in the previous assay, including H37Rv. In all cases, the infection by MTB increased the TNF-α production compared to the values observed in the non-infected control. The TNF-α production dynamics along the infection differed among the isolates analyzed. Four isolates induced a peak level of TNF-α at day 1 after infection, and this was followed by a rapid decrease in secretion (Figure 3A). The other two isolates were those that had the highest growth rates in the THP-1 assay. In these isolates, TNF-α production followed a different pattern, namely, production of TNF-α was contained from the start of infection, and was significantly lower than that induced by the remaining isolates at day 1 after infection. TNF-α levels in these isolates continued to decrease throughout the infection (Figure 3A).

The IL-10 secretion profiles in THP-1 cells infected with all the Beijing isolates were similar to the non-infected controls at early stages of infection; IL-10 production was not detected up to day 1 after infection (Figure 3B). However, from this point, the IL-10 production dynamics differed among the isolates analyzed with peak levels occurring at day 4. IL-10 levels subsequently decreased in all of the isolates except two, in which production increased until infection resolved (Figure 3B). These two isolates corresponded to those which contained production of TNF-α and showed the highest growth rates in THP-1-infected cells. Thus, a correlation was found between intracellular replication, production of TNF-α, and the immunoregulatory response through IL-10 in THP-1 infected cells.

Both of the strains with a differential behaviour in the THP-1 model corresponded to South American orphan cases: one belonged to phylogenetic group 4 and was isoniazid-resistant, and the other belonged to phylogenetic group 3 and was pan-susceptible and displayed the lowest number of IS*6110 *copies (nine).

The Beijing isolate responsible for the TB outbreak on Gran Canaria Island was not distinguishable from other isolates. It had an average intracellular growth rate and did not control TNF-α levels at early stages of the infection.

When we considered the cluster/orphan status of the isolates, analysis of intracelullar growth rates and cytokine expression profiles did not reveal a correlation between cluster/orphan status and infective behaviour in the THP-1 model.

## Discussion

The worldwide distribution of the Beijing lineage has been well documented [6-8], being this genotype highly prevalent (70-80% in total isolates) in East Asia (China, Korea, Japan, etc). However, the proportion of Beijing strains in Western Europe is low. In two countries of the Mediterranean area, Italy and Spain, the marked increase in the number of immigrants in recent years has led to an increase in the numbers of TB cases that can be attributed to imported strains. In Madrid (Spain) and Tuscany (Italy) during the period 2004-2006, slightly more than 40% of all cases of TB were detected in immigrants [15,21]. We characterized the genotypic and phenotypic features of the Beijing lineage in a setting where it is not frequently isolated and where it is mostly detected in immigrant cases.

Spoligotyping, sequencing of *pks15/1*, and analysis of the presence of the RD105 region revealed a low representativeness of this lineage in our population, as previously described in Central and Western Europe [8,9,22]. These studies also showed that Beijing strains in our area are mainly found in immigrants (ie, around 85% of our isolates were from immigrants, mostly Peruvians and Ecuadorians). This is consistent with the results of studies which report that the Beijing lineage was also imported to Europe via South America [23,24].

The Beijing lineage is generally considered to be associated with drug-resistant phenotypes, although this may not be true for all geographic settings [7,8] and most of the Beijing strains in our study were susceptible. In fact, drug-resistant but also pan-susceptible strains have been associated with TB outbreaks [25] and it has recently been proposed that mainly atypical variants of Beijing strains are those linked to resistance [26].

IS*6110*-RFLP based genotyping was performed in order to establish a molecular epidemiological profile for the Beijing strains in the Spanish sample. Nineteen representative patterns of the Beijing genotype have been reported, and most of them have a high IS*6110 *copy number (15-26) [6,27]. The wider range of IS*6110 *copy numbers-- 9 to 22--alerts to the existence of Beijing isolates without a high number of IS*6110 *copies. The RFLP patterns of a 5-year population based sample enabled us to define two clusters including 7 of the 26 Beijing isolates of the study (26.9%); the first cluster contained three Ecuadorian immigrants and an autochthonous case, and the second contained three autochthonous patients. The members of the latter group shared an identical IS*6110*-RFLP pattern with the one involved in the TB outbreak on Gran Canaria Island in the 1990s [14]. In our study, MIRU-15 was less discriminatory (13 of 26 isolates in five clusters) than RFLP. Recently, new hypervariable loci have been evaluated to increase the discriminatory capacity of the 15-loci VNTR typing method in the Beijing lineage [19,20,28,29]. A selection of them together with the 15-loci set, increased the discriminatory power to values even higher than those of RFLP.

The distribution of the Beijing lineage in different geographic areas and its ability to disseminate suggest that this phylogenetic lineage is better adapted to infect and cause TB in humans than other genetic lineages of MTB. It has been associated with high virulence and rapid growth in both *in vitro *and *in vivo *infection models [10,11,30]. These features are considered to be behind the success of Beijing strains, which is a consequence of their control over the immune response [12].

We attempted to characterize the infective features of the Beijing isolates in our sample by assaying a selection of isolates. We enriched the sample to be assayed in the infectivity model with additional Beijing isolates from another setting (Tuscany, Italy) in the Mediterranean area that had features, namely clustered strains, which were underrepresented in our area. As the Beijing lineage was the only genotype showing a steady expansion in Tuscany with frequent clustering (involving immigrants and autochthonous patients) [15], we included several isolates from this area in our sample.

To characterize the infective features of the Beijing isolates, an *in vitro *infection model using differentiated THP-1 cells was applied, which has been considered a good macrophage model [31-35] and which validity was proved after demonstrating that THP-1 cells differentiated with PMA express CD14, an antigen considered a marker for macrophages [36]. This model is also a good alternative for evaluation of the infectivity of MTB [10,37,38].

Although the number of isolates in our study is small to draw general conclusions, an interesting finding was that the isolates showed heterogeneous infective behaviour, with a wide range of intracellular growth rates. Two isolates showed the highest growth rates and stood out significantly from the others.

We tested cytokine production in the *in vitro *infections, focusing on TNF-α and IL-10 as the main representatives of the Th-1 and Th-2 responses. In our model, the levels of cytokines always increased after infection, indicating that the assay, although activated by the addition of PMA, is not saturated. It allowed a measuring window to identify different infective behaviours among the strains analyzed. Indeed, it allowed us to efficiently measure the increases, in or maintenance or contention of cytokine production after infection caused by specific strains, which was our aim. We observed that infection with the isolates with the highest intracellular replication levels elicited the lowest TNF-α levels in THP-1-infected cells. At early stages of infection, these isolates induced significantly lower TNF-α production than the other isolates, and maintained this level until the end of infection, thus indicating failure to correctly induce the cytokine-dependent Th1-type protective immune response. Other authors have also observed a wide range of intracellular replication rates among Beijing isolates and an inverse association between intracellular replication levels and TNF-α production [30,39].

Furthermore, low-virulence strains are associated with a more vigorous immune response with high levels of type 1 cytokines (TNF-α, IFN-γ, IL-12) [10,13,40]. These data suggest that the infective advantage of Beijing strains should not be considered as an intrinsic feature of the lineage, but as a characteristic of certain representatives. These findings are highly relevant, as the outcome of the infection is related to the ability of MTB to regulate the induction of cytokines that are essential for the development of an efficient immune response [41]. As shown by our study and others, the virulent Beijing representatives induced high production of proinflammatory cytokines, which is quickly controlled, thus decreasing their levels and giving rise to a more effective infection. Phenol glycolipid (PGL), has recently been proposed as a virulence factor in Beijing strains [12]. This molecule can inhibit the release of key inflammatory effector molecules *in vitro *and has been considered responsible for the hypervirulent phenotype of Beijing strains, both in murine and rabbit infection models [12,42]. The different sub-groups of the Beijing lineage have recently been shown to contain different percentages of PGL-producing strains [18]; therefore, other factors could determine the hypervirulence of certain Beijing strains. As most of the isolates in our study belonged to sub-group 3, it was not possible to explore in depth the relationships between infectivity and PGL production. However, isolates belonging to sub-group 3 displayed different intracellular growth rates. The only representative belonging to sub-group 4 (with the highest percentage of PGL-producing strains) showed the highest intracellular replication levels. Therefore, according to Reed *et al *[18], it would be very interesting to evaluate PGL production in these isolates to determine whether their hypervirulent phenotype (high intracellular replication rates, low production of TNF-α) could correlate with the synthesis of this complex glycolipid.

Some studies have analyzed the relationship between intracellular growth and transmissibility [40,43], and concluded that the extensive spread of an MTB strain correlated with its high capacity to replicate, which is considered a marker of virulence. We studied intracellular replication levels of clustered and orphan strains, and observed no significant differences in growth rates or TNF-α production. In addition, the two isolates with the highest growth rates were orphan isolates. Therefore, virulence features are not always associated with clustered/orphan status.

Clustered/orphan status could be a consequence of bacterial factors, although epidemiological features must also be taken into consideration. In this sense, it is interesting to mention our findings for the isolates corresponding to the strains involved in the Gran Canaria outbreak. The three patients infected with this strain had lived on Gran Canaria Island before arriving in Madrid, and there was a three-year interval between each diagnosis. This cluster seems more likely to be a coincidental finding in Madrid of three cases infected on Gran Canaria Island than a recent transmission in Madrid. Other than these cases, no secondary cases involving this genotype have been found since the last one (2006), which is the opposite of the explosive spread of the same strain on Gran Canaria Island. The lack of secondary cases by this genotype after its first detection in Madrid could suggest that epidemiological features more than bacteriological features (virulence, transmissibility, infectivity) could have been responsible for the Gran Canaria outbreak. Consistent with this explanation, the representative of this cluster did not show any replicative advantage or control over the immune response, and, therefore, this strain should not be considered especially virulent or transmissible.

## Conclusions

In summary, we provide an outline of the genotypic and infective features of Beijing isolates identified in Spain and Italy. We show the low representativeness of this lineage in the study population, the association between the lineage and immigration, and the lack of association with resistant phenotypes. The infective profile of the Beijing isolates was markedly heterogeneous, suggesting the existence of certain highly virulent representatives in a non-homogeneous lineage. In our sample, we did not find a correlation between virulence and phylogenetic group or resistance. A correlation between *in vitro *infectivity and the clustered/orphan status of the isolates was not found, which could reflect the complex process that infection/transmission is with a potential role for patient-related factors (economical, social, epidemiological aspects). The Beijing strain which was extensively transmitted on Gran Canaria Island displayed a completely different epidemiological behaviour in Madrid, and did not show a highly infective phenotype *in vitro*. Various factors, both bacterial and epidemiological, seem to be behind the success and higher prevalence of Beijing strains compared with the other genetic lineages of MTB. The specific role that these factors could play in different contexts must be clarified before establishing general assumptions about the Beijing lineage.

## Methods

### MTB strains

Clinical samples were processed according to standard methods and grown in Lowenstein-Jensen slants (Becton Dickinson, Sparks, Maryland, USA). During the period 2002-2007, we received for genotyping a total of 2391 MTB cultures from two population-based studies in Spain between 2004 and 2008 [44,45]: 1872 isolates were from five urban areas in Madrid (6,081,689 inhabitants) and 519 were from Almeria (south-eastern Spain 646,633 inhabitants).

We also included, exclusively for the infectivity assays, eight Beijing isolates from a previous study performed from 2002 to 2004 in Tuscany (central Italy, 3,600,000 inhabitants) [15].

### Identification and genetic characterization of Beijing strains

Spoligotyping was performed following the manufacturer's instructions (Isogen, Netherlands). The Beijing genotype was assigned on the basis of the spoligotype. In particular, isolates with spoligotype patterns characterized by deletion of spacers 1-34 were defined as "typical" Beijing, whereas isolates with additional deletion of one or more of the last nine spacers were defined Beijing-like according to the criteria of the international database SpolDB4 [22].

To confirm this identification of Beijing isolates by spoligotyping and also to refine the genetic characterization of the Beijing isolates, the *pks15/1 *gene and thegenomic deletions RD105, RD181, RD150, and RD142 were analyzed.

An intact *pks15/1 *gene has been reported to be a marker of the Beijing lineage [4,17], whereas a 7-bp deletion has been found for non-Beijing isolates. We purified DNA using standardized methods [46] to verify the marker by amplification and DNA sequencing [4] using an ABI-PRISM 310 sequencer (Lab Centraal B.V., Haarlem, NL).

The genomic deletions RD105, RD181, RD150, and RD142, which sub-classify the Beijing lineage, were identified by PCR using primers and conditions described elsewhere [5].

### Molecular typing methods

DNA extraction and restriction fragment length polymorphism typing with the insertion sequence IS*6110 *(IS*6110*-RFLP) were performed according to standard methods [46]. Computer-assisted analysis of IS*6110 *fingerprints was carried out using Bionumerics 5.1 software (Applied Maths, Kortrijk, Belgium). Mycobacterial interspersed repetitive unit-variable number tandem repeat typing (MIRU-VNTR) was performed by amplifying the 15 MIRU-VNTR loci as described elsewhere [47], with some modifications [48]. The number of repetitions in each locus was calculated by applying the corresponding conversion tables (P. Supply, personal communication). The MIRU type was defined after combining the results for the 15 loci in the following order: MIRU4, MIRU26, MIRU40, MIRU10, MIRU16, MIRU31, Mtub04, ETRC, ETRA, Mtub30, Mtub39, QUB4156, QUB11b, Mtub21, and QUB26.

Five additional loci were added to the MIRU15 set: QUB11a and QUB18 [19,20], QUB3232 [19], and VNTR3820 and VNTR4120 [28,49], which were selected based on the high polymorphism found for the Beijing clade when applying these loci [28]. They were all amplified using simplex polymerase chain reaction (PCR) with primers described elsewhere [19,28,50]. Dimethyl sulfoxide (Sigma-Aldrich) was added in the amplification reactions for VNTR3820 and VNTR4120 (8%) and QUB11a, QUB18, and QUB3232 (12%). The sizes of the PCR products were calculated after electrophoresis in 2% agarose gels (MS8 agarose; Pronadisa, Madrid, Spain) for 17.5 hours at 45 V (for products under 800 bp) or 22 hours (for larger products). Assignation of alleles was based on table sizes kindly provided by Dr. Tomotada Iwamoto (Microbiology Dep., Kobe Health Institute, Japan) and on data published elsewhere [19,20,28,49].

In certain cases, the large size for some products obtained at loci QUB11a, VNTR3820, and QUB3232 did not allow accurate assignation of alleles. In these cases, we only could estimate that the number of repetitions was higher than 20 (> 20). When we observed products differing in size in groups of isolates with more than 20 repetitions, we sub-labeled them > 20a, > 20b, > 20c and > 20d.

For the analysis by MIRU-VNTR of the isolates sharing RFLP pattern with the strain involved in the Gran Canaria outbreak (analyzed in Hospital Miguel Servet, Zaragoza), only the 15-loci format was applied and not the expanded set of five additional loci, because these have not been validated for interlaboratory comparisons due to low interlaboratory reproducibility.

### Cluster analysis

Genotypic patterns were analyzed using Bionumerics 4.6 (Applied Maths, Belgium). Dendrograms were generated using the unweighted pair group method with arithmetic averages (UPGMA) and the Dice coefficient or the categorical coefficient for IS6110-RFLP and MIRU-15 analysis, respectively. RFLP clusters and orphan status were defined for isolates sharing identical fingerprints after analyzing the patterns for the 2391 MTB isolates from the population-based sample. MIRU clusters were defined for isolates sharing identical patterns.

### Susceptibility test

Susceptibility testing with isoniazid, rifampin, streptomycin, pyrazinamide, and ethambutol was performed using the mycobacterial growth indicator SIRE system (Becton Dickinson, Sparks, Maryland, USA).

### Cell cultures

The human promonocytic cell line THP-1 was obtained from the American Type Culture Collection (TIB-202; Manassas, Virginia, USA). Cell cultures were maintained in modified RPMI 1640 + L-glutamine (Gibco, Grand Island, NY) supplemented with 10% fetal bovine serum (Gibco, Grand Island, NY), 10 mM HEPES, and 50 μg/ml gentamicin (Gibco, Grand Island, NY). Cultures were maintained at 7-10 × 10^5 ^cells/ml and incubated at 37°C in 5% CO_2 _in a humidified incubator. In order to ensure that we are working with a macrophage model, THP-1 cells were differentiated to adherent macrophages by the addition of 200 nM phorbol myristate acetate (PMA) (Sigma, St. Louis, MO) for 3 days at 37°C in 5% CO_2_.

### Cell infection

Cells were infected as described elsewhere [10], with slight modifications. Briefly, differentiated THP-1 cells seeded in 24-well flat-bottom tissue culture plates were washed and the medium was replaced to remove PMA and gentamicin 2 hours before the addition of bacteria. Cells were infected with a multiplicity of infection of 2-10 bacteria per cell and incubated for 3 hours at 37°C in 5% CO_2_. After incubation, monolayers were thoroughly washed with phosphate-buffered saline to remove extracellular bacteria and fresh medium was added. To evaluate the bacterial growth, supernatants were aspirated and monolayers were lysed with 0.5% Nonidet P40 (Roche Diagnostics, Mannheim, Germany) at 3 hours and days 1, 4, and 7 after infection. Serial 10-fold dilutions of cellular lysates were plated on Middlebrook 7 H11 plates and incubated for 3 weeks at 37°C in 5% CO_2_, and colonies were counted. Intracellular growth was expressed as the growth rate, which is the slope of the function of log_10 _CFU values throughout the infection period (3 hours and days 1, 4, and 7). Three or more independent experiments were performed for each assayed strain.

### Cytokine analysis

Culture supernatants from control and infected THP-1 cells were harvested after 3 hours and on days 1, 4, and 7, frozen at -70°C, and assayed using an enzyme-linked immunosorbent assay (ELISA) kit according to the manufacturer's instructions (BD Biosciences, Lincoln Park, NJ) to measure levels of tumor necrosis factor alpha (TNF-α) and interleukin 10 (IL-10).

### Statistical analysis

Three independent experiments were performed per strain. The means and standard errors were determined for each measurement in both intracellular growth and cytokine production. One-way analysis of variance with repetitive measures was used to determine P values, which were adjusted using the Bonferroni method. All the comparisons were carried out using the program SPSS 17.0.

## Competing interests

The authors declare that they have no competing interests.

## Authors' contributions

MA designed and performed all the experiments related to pks15/1, RDs and infectivity assays, analyzed the results, produced the first version of the MS and was involved in the correction of the MS. NA performed the molecular-epidemiology study, analyzed the results and collaborated in the production of the first version of the MS. CG provided a selection of MTB strains from Tuscany, Italy and critically reviewed the final version of the MS. MML and members from the INDAL-TB group, coordinated the molecular epidemiological study in Almeria. MH performed the IS6110-RFLP and spoligotyping assays and analyzed the results. SS obtained and provided the IS6110-RFLP and MIRU-15 data for the Beijing isolates involved in the outbreak of G. Canaria and collaborated in the comparative analysis of these data with those obtained in Madrid. MJRS performed all the microbiological procedures. EB critically reviewed the final version of the MS. DGV designed the study, supervised all the experimental work, analyzed the results, corrected and produced the final version of the MS.

All the authors read and approved the final version of the MS
